# Case of Incidental Asymptomatic Carotid Body Paraganglioma in a Patient with Infectious Mononucleosis

**DOI:** 10.7759/cureus.5443

**Published:** 2019-08-20

**Authors:** Eric L Cox, Anthony Diponio, David Seel

**Affiliations:** 1 Otolaryngology, Beaumont Health, Farmington Hills Campus, Farmington Hills, USA; 2 Otolaryngology, Michigan State University College of Osteopathic Medicine, East Lansing, USA

**Keywords:** paraganglioma, tonsillectomy, mononucleosis

## Abstract

A paraganglioma is a rare form of neuroendocrine tumor of neural crest cell origin. These tumors arise from autonomic ganglia, and are classified based on their origin in the autonomic nervous system, with sympathetic paragangliomas being more commonly located in the abdomen and parasympathetic paragangliomas more commonly found in the head and neck region. Paragangliomas have a characteristically slow growth rate, and while many will present due to symptoms of mass effect or from hyper functional catecholamine secretion, some tumors are diagnosed incidentally on imaging studies. In this report, we present the case of a 33-year-old man who presented to the emergency department with symptoms of infectious mononucleosis and recurrent tonsillitis, for which he was scheduled to have a tonsillectomy in the near future, but was incidentally found on a CT scan of the neck to have an asymptomatic carotid body paraganglioma. Due to the close anatomical relationship between the carotid artery and tonsillar bed, had this surgery occurred without first discovering the paraganglioma it may have placed the patient at increased risk for a disastrous intraoperative hemorrhage. This idea prompts otolaryngologists to consider how best to approach the diagnosis of paragangliomas in patients that have yet to become symptomatic.

## Introduction

Paragangliomas are a rare type of highly vascular neuroendocrine tumor of neural crest cell origin that arise from autonomic ganglia, comprising about 1 in 100,000 of all tumors in the head and neck region. These tumors are commonly compared to pheochromocytomas, also known as “intra-adrenal paragangliomas”, due to their ability to secrete catecholamines, although recent literature suggests only 1-3% have this ability [1]. Paragangliomas can be broken down into two categories based on their origin: sympathetic and parasympathetic ganglia. Sympathetic paragangliomas are generally found in the abdomen, and are more likely to secrete catecholamines, however, parasympathetic gangliomas are found almost exclusively in the head and neck, and most commonly arise from the carotid body or jugulotympanic paraganglia [2]. These usually present as incidental findings or from symptoms of mass effect due to the fact that they are generally benign and rarely secrete catecholamines.

Carotid body paragangliomas are typically slow growing in nature, with a delay between first symptoms and time of diagnosis of approximately 4-7 years. They typically present as a lateral neck mass, which is mobile laterally but not cranio-caudally, referred to as a positive Fontaine sign. More extensive paragangliomas can present as a pulsatile mass, may have cranial nerve involvement, and may even present with carotid sinus syndrome, which refers to loss of consciousness with reflexive bradycardia and hypertension. Despite all of this, carotid body paragangliomas usually present as incidental findings or are discovered inadvertently on radiographic imaging. Typically, these tumors can be identified by computed tomography (CT) of the neck with contrast or, more commonly, by magnetic resonance images (MRI) with gadolinium. In this report, we present the case of a 33-year-old man who presented to the emergency department with cervical lymphadenopathy and a sore throat, and was incidentally found on a neck CT scan to have an asymptomatic carotid body paraganglioma.

## Case presentation

Our patient was a 33-year-old male who presented with a five-day history of fever, generalized body aches, sore throat, odynophagia, nausea, vomiting, dizziness, and diffuse abdominal pain. Past medical history was significant for gastroesophageal reflux and recurrent episodes of tonsillitis, for which the patient was scheduled for a tonsillectomy.

**Figure 1:**
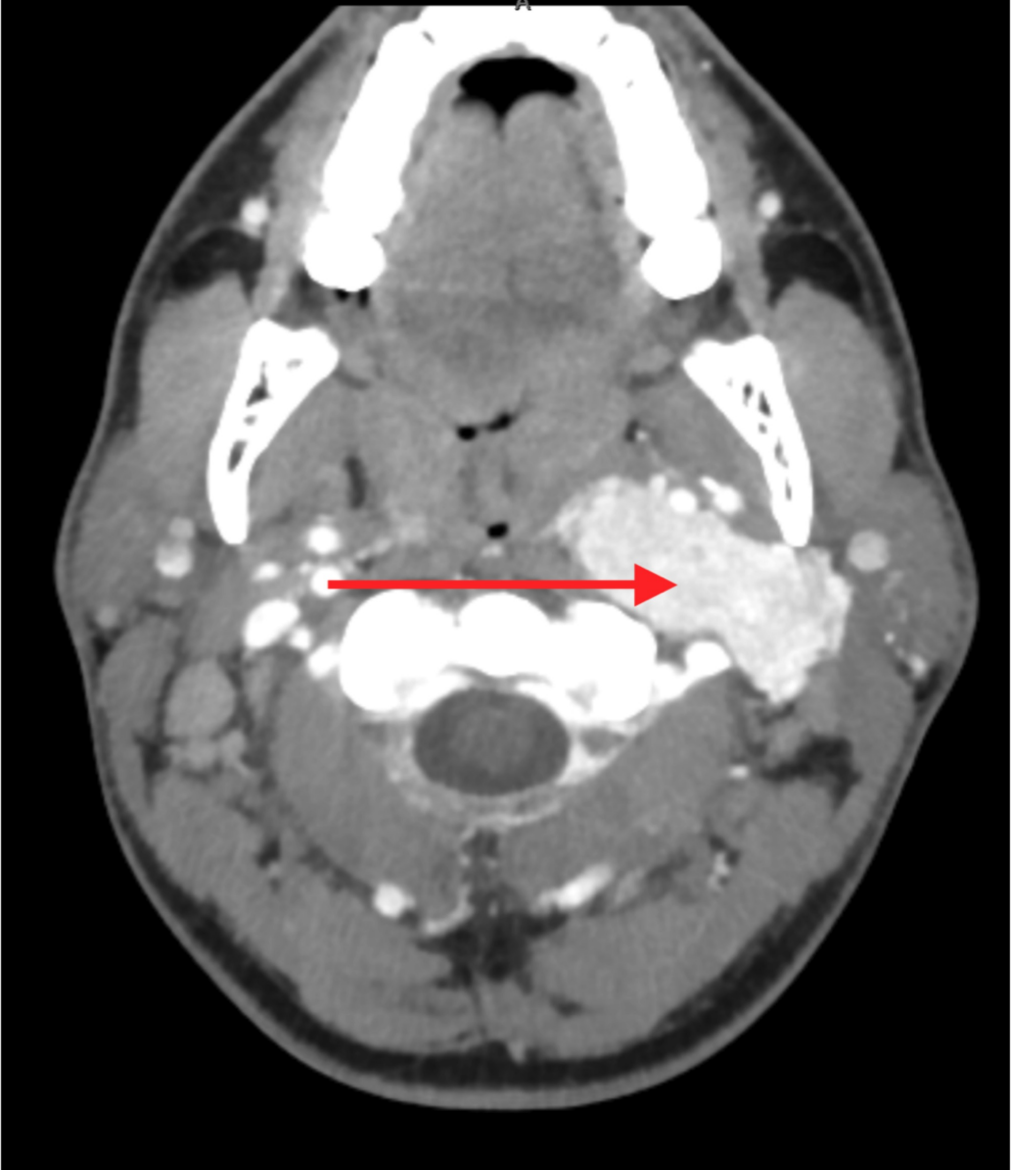
CT neck with IV contrast (axial view). Prominent pharyngeal and adenoidal tonsils with bilaterally enlarged cervical chain and submental lymph nodes. Large avidly enhancing mass within left carotid sheath.

**Figure 2:**
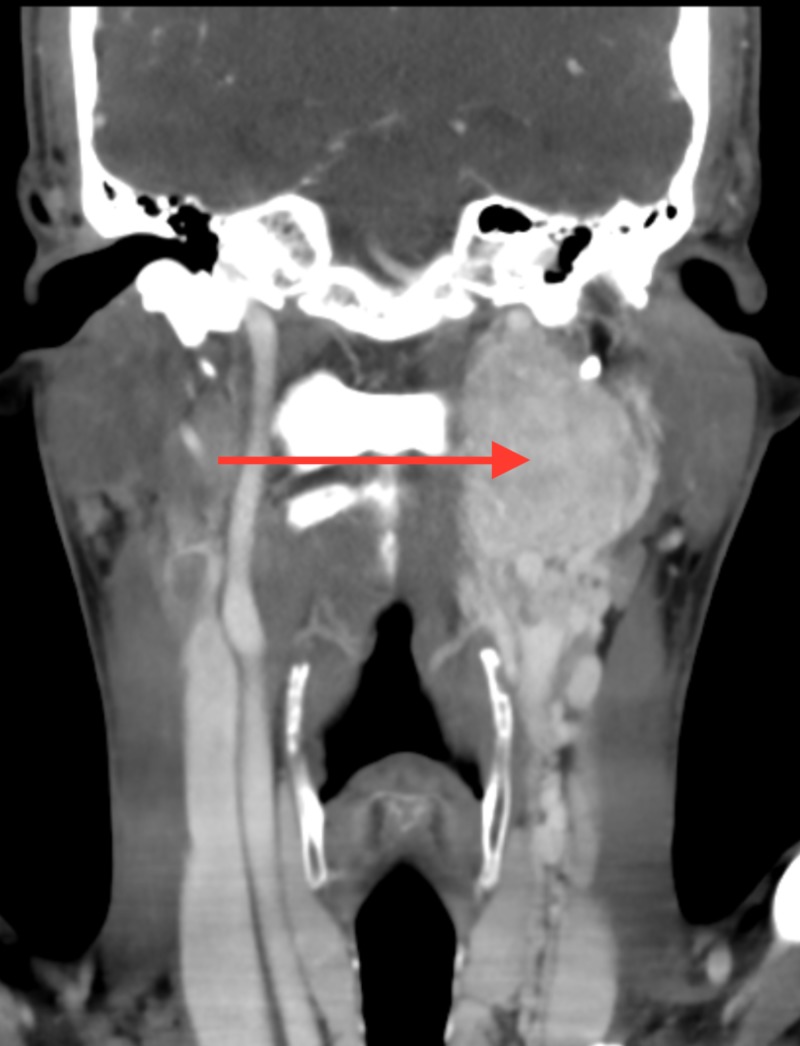
CT neck with IV contrast (coronal view). Large avidly enhancing mass within left carotid sheath extending from level of hyoid to jugular foramen at skull base.

Upon arrival to the emergency center, he was found to have a blood pressure of 144/102 mm/Hg, heart rate of 110 BPM, fever of 39 C, and a WBC count of 5,400/mm^3^. On physical exam, tonsils were significantly enlarged (graded 4+ on a scale of zero to four) and erythematous, and he was noted to have enlargement of his left peritonsillar area concerning for an abscess, while the remainder of the physical exam was unremarkable. A Monospot test obtained in the emergency department was positive, indicating a primary infection with infectious mononucleosis. Due to the patient’s history of recurrent tonsillitis, fever, and asymmetric tonsillar hypertrophy, a CT scan of the neck with IV contrast was also ordered. This resulted in an incidental finding of a large avidly enhancing mass within the left carotid sheath that extended from the level of the hyoid to the jugular foramen at the skull base, in addition to prominent pharyngeal and adenoidal tonsils, with bilaterally enlarged cervical chain and submental lymph nodes (Figures 1, 2). Based on its location and characterization, this mass was deemed likely to be a carotid body paraganglioma, but required further investigation with a neck MRI and magnetic resonance angiography (MRA) per radiology recommendations. Plasma and urine catecholamines and metanephrines were also ordered, which were within normal limits.

**Figure 3:**
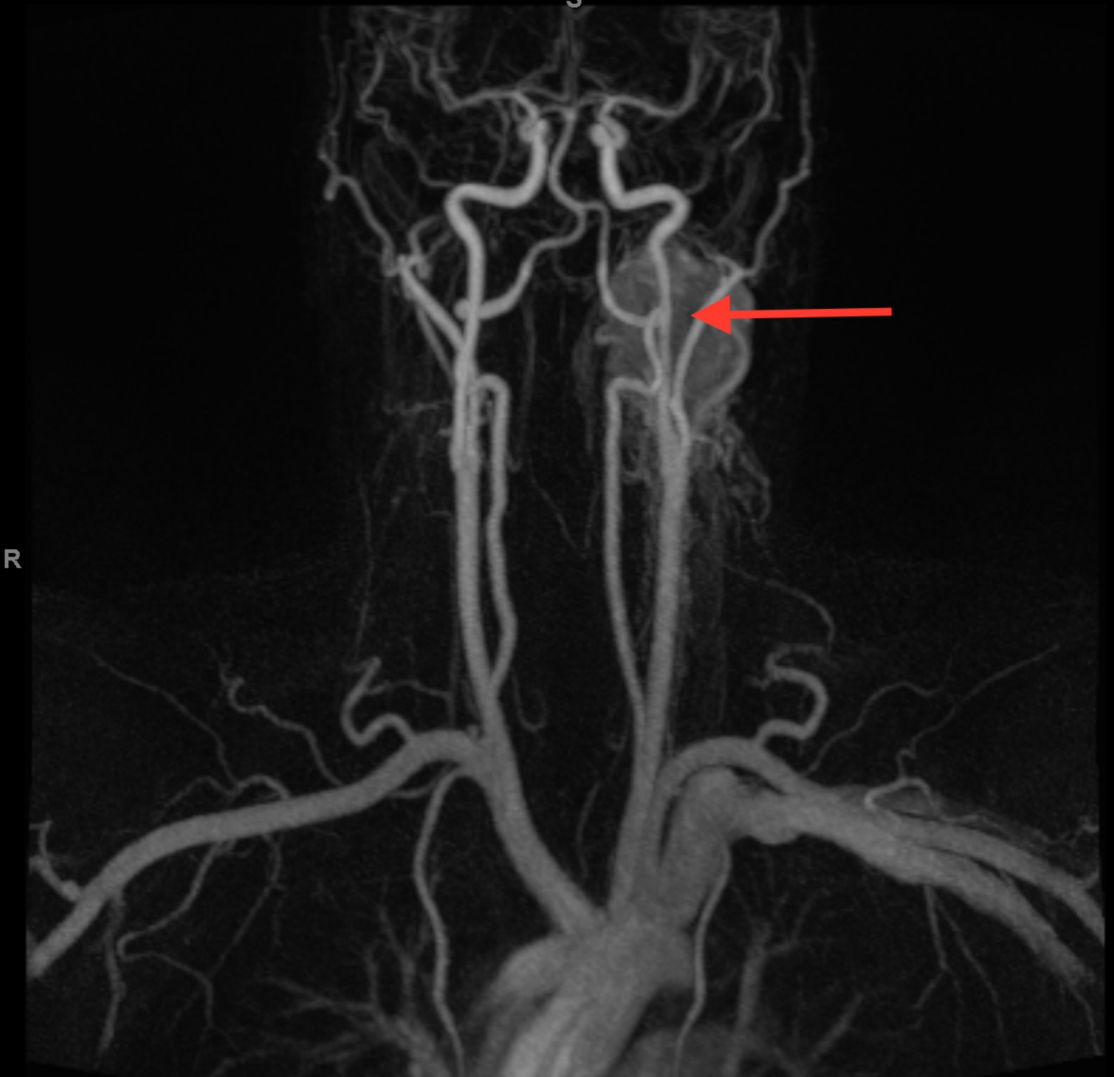
Magnetic resonance angiography with and without gadolinium (coronal view). Left neck mass displacing the left internal and external carotid arteries, without significant luminal narrowing. Extensive vascularity of the mass from branches of the external carotid artery.

An MRI of the neck with and without IV gadolinium re-demonstrated the enhancing left neck mass measuring 5 x 5 x 2 cm most consistent with a paraganglioma. A magnetic resonance angiography of the carotid arteries and neck vascularity re-demonstrated this left neck mass, and the mass was shown to displace the left internal and external carotid arteries without any luminal narrowing. There was extensive vascularity of the mass, being fed by multiple branches of the external carotid artery (Figure 3).

After admission and treatment, the patient was discharged home and instructed to follow up outpatient with both ENT and neurosurgery to discuss medical and surgical options for removal of the paraganglioma.

## Discussion

Carotid body paragangliomas have a characteristically slow growth rate that tends to delay the initial diagnosis and presentation. Along with presenting as a lateral neck mass, these tumors can secrete catecholamines, cause cranial nerve disturbances, and can even be associated with an audible bruit or pulsatile mass. Despite these tumors’ potential ability to secrete catecholamines, it was not until 1962, when Glenner et al. described the first norepinephrine secreting carotid body tumor. Most recent literature suggests that although these tumors have neurosecretory granules, only 1 to 3% are found to be functional, with a recent retrospective study by Smith et al. reporting a 4.6% rate, which again brings into question how exactly these tumors are discovered [3]. In one study of 236 patients with 297 benign paragangliomas at the Mayo Clinic during 1978-1998, 9% of these patients were diagnosed incidentally on imaging studies [4]. A total of 205 out of the 297 tumors were located in the head and neck, and 10% of these were discovered incidentally on imaging studies while 4% were hyperfunctional. A majority of these tumors were discovered due to symptoms of local mass effect (tinnitus, neck mass, cranial nerve dysfunction) [4]. Out of the 92 tumors located below the neck, 27% were discovered incidentally from imaging studies, while 43% were hyperfunctional [4]. From this data it can be concluded that most paragangliomas are located in the head and neck, and while it is more common for tumors below the neck to be hyperfunctional, all patients with paragangliomas must be screened for catecholamine excess.

Several physical exam signs and symptoms have been described related to the compression nature of head and neck paragangliomas. Eponyms for cranial nerve palsies have been described, such as Vernet syndrome (cranial nerve palsy of IX - XI), Collet Sicard syndrome (cranial nerve palsy of IX - XII), and Villaret syndrome (cranial nerve palsy of IX - XII and sympathetic chain defects). Given the neurologic nature of a majority of these physical exam findings attributed to paragangliomas, it is imperative for the examiner to perform a thorough neurologic exam in conjunctive with the history and physical.

Historically, carotid body paragangliomas have been treated via primary surgical resection, with possible preoperative embolization. Other literature suggests that primary radiation may be used for nonoperative candidates, advanced tumors, or if there is presence of residual disease. A study published by Hu and Persky in 2016 suggested that observation without treatment is a viable option for patients who are asymptomatic, and specifically for older patients with significant comorbidities [5]. The study also suggested that first-line surgery is preferred for patients with carotid body tumors, especially if the tumor is less than 5 cm and lacks carotid artery encasement [5]. Most literature suggests that regardless of the type of paraganglioma, a multidisciplinary approach should be undertaken if primary surgical intervention is warranted, with emphasis on single-modality therapy that will offer optimal outcomes.

In our case of this 33-year-old male patient, urine and plasma metanephrines and catecholamines were ordered, but were found to all be within normal limits. His physical examination failed to reveal any neurologic deficits or other physical exam findings that may be contributed to the compression nature of most paragangliomas. He had no airway compressive symptoms or other mass effect symptoms, as well as no outwardly apparent lateral neck mass. However, had the patient not presented to the ED with symptoms of infectious mononucleosis and tonsillitis, this tumor, with its rich vascular supply, may have continued to grow and eventually cause symptoms of mass effect or may have even led to catastrophic vascular injuries during the patient’s scheduled tonsillectomy. The close anatomical relationship between the carotid artery and the tonsillar bed, most frequently cited as lying 2-3 cm deep to the superior pharyngeal constrictor, lends credence to the tenuous nature of his tonsillectomy, had it occurred.

## Conclusions

The presented case in this paper highlights the silent nature of most of these tumors, and sparks thought as to how best to approach diagnosis of paragangliomas in the head and neck region that have yet to produce any discernable physical exam findings. At very least, this is a case of divine providence, where an asymptomatic paraganglioma was incidentally discovered when a patient presented to the emergency department complaining of an entirely separate etiology.
